# Secure quantum remote state preparation of squeezed microwave states

**DOI:** 10.1038/s41467-019-10727-7

**Published:** 2019-06-13

**Authors:** S. Pogorzalek, K. G. Fedorov, M. Xu, A. Parra-Rodriguez, M. Sanz, M. Fischer, E. Xie, K. Inomata, Y. Nakamura, E. Solano, A. Marx, F. Deppe, R. Gross

**Affiliations:** 1 Walther-Meißner-Institut, Bayerische Akademie der Wissenschaften, 85748 Garching, Germany; 2 Physik-Department, Technische Universität München, 85748 Garching, Germany; 3 Department of Physical Chemistry, University of the Basque Country UPV/EHU, Apartado 644, E-48080 Bilbao, Spain; 4 Munich Center for Quantum Science and Technology (MCQST), Schellingstr. 4, 80799 Munich, Germany; 5 RIKEN Center for Emergent Matter Science (CEMS), Wako, Saitama 351-0198 Japan; 6 National Institute of Advanced Industrial Science and Technology, 1-1-1 Umezono, Tsukuba, Ibaraki 305-8563 Japan; 7 Research Center for Advanced Science and Technology (RCAST), The University of Tokyo, Meguro-ku, Tokyo 153-8904 Japan; 8 IKERBASQUE, Basque Foundation for Science, Maria Diaz de Haro 3, 48013 Bilbao, Spain; 9 Department of Physics, Shanghai University, 200444 Shanghai, China

**Keywords:** Superconducting devices, Quantum physics, Quantum information

## Abstract

Quantum communication protocols based on nonclassical correlations can be more efficient than known classical methods and offer intrinsic security over direct state transfer. In particular, remote state preparation aims at the creation of a desired and known quantum state at a remote location using classical communication and quantum entanglement. We present an experimental realization of deterministic continuous-variable remote state preparation in the microwave regime over a distance of 35 cm. By employing propagating two-mode squeezed microwave states and feedforward, we achieve the remote preparation of squeezed states with up to 1.6 dB of squeezing below the vacuum level. Finally, security of remote state preparation is investigated by using the concept of the one-time pad and measuring the von Neumann entropies. We find nearly identical values for the entropy of the remotely prepared state and the respective conditional entropy given the classically communicated information and, thus, demonstrate close-to-perfect security.

## Abstract (web summary)

Continuous-variable remote state preparation in the microwave domain would allow to leverage the superconducting technology for quantum networks applications. Here, the authors show how to deterministically prepare squeezed Gaussian states across 35 cm using previously shared entanglement.

## Introduction

In quantum technology, an efficient and secure exchange of quantum information between quantum nodes plays a crucial role^1^. One of the first protocols realizing such a task was quantum teleportation, where an unknown quantum state is safely transferred from one party to another by using a shared entangled resource and classical feedforward^2,3^. In a different scenario, where one party has full classical knowledge about a to-be-communicated quantum state, remote state preparation (RSP) can be used to remotely create this quantum state by employing similar tools as in quantum teleportation^4–7^. Compared to known classical methods, both protocols provide a quantum advantage as they require a smaller amount of classical information in the feedforward signal in order to communicate a desired quantum state^8^. However, in contrast to quantum teleportation, RSP allows for a nontrivial trade-off between the amount of classical communication and entanglement necessary for a successful protocol^6^. Furthermore, the use of an entangled resource allows RSP to operate perfectly secure^8^. Even though RSP is extensively investigated both theoretically and experimentally for discrete-variable systems^9–11^, deterministic implementations with continuous-variable systems are still lacking^12,13^. At the same time, quantum communication based on continuous-variables is a field of intense research^14,15^ investigating, e.g., quantum key distribution^16^, quantum teleportation^17,18^, dense coding^19^, and free-space quantum communication^20^.

Quantum communication in the microwave domain is motivated by the tremendous progress in the area of quantum information processing with superconducting circuits. In particular, the development of superconducting multi-qubit processors^21,22^, operated at gigahertz frequencies has been highly successful. We promote an approach of quantum communication directly in the microwave regime based on propagating squeezed states. Since these states have the same frequency and are generated by technology platforms already used for superconducting quantum computers, there is no mismatch between communication and data processing units. This approach is expected to be useful for short and medium distances, where superconducting waveguides can be used.

In this work, we realize deterministic continuous-variable RSP by creating Gaussian squeezed states with tunable squeezing level and squeezing angle over a distance of 35 cm. We investigate the phase space of remotely preparable squeezed states and obtain good agreement with our model calculations based on the input-output formalism. Additionally, we find that our scheme corresponds to an extension of the one-time pad cryptographic protocol^23^ into the quantum regime which allows for information-theoretic security. In contrast to already demonstrated quantum state transfer protocols between superconducting circuits^24,25^, our protocol does not directly transmit the target states to the receiving party. Moreover, it can be operated in the continuous regime and utilizes preshared entanglement to enable secure communication between parties. Since the generation and manipulation of Gaussian states is well understood^14^, they offer a viable option for building future intracity low-temperature quantum networks^26^.

## Results

### RSP with squeezed microwaves

**Fig. 1:**
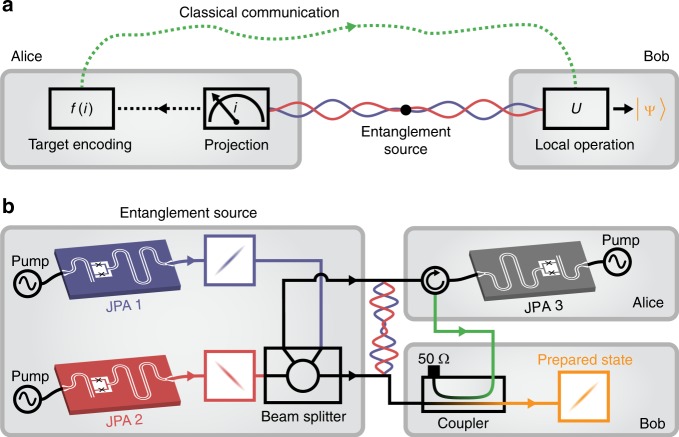
Principle of remote state preparation. **a** General RSP scheme: Alice remotely prepares a desired state at Bob’s side using a quantum resource and classical communication (feedforward). **b** Experimentally implemented RSP scheme: a two-mode squeezed state (left) serves as quantum resource and the feedforward to Bob (right) is implemented using JPA 3 and a directional coupler

The general idea behind the RSP protocol and our experimental implementation using continuous-variable microwave states are described in Fig. 1. We use flux-driven Josephson parametric amplifiers (JPAs) as the key elements for the generation and manipulation of squeezed microwave states^27–29^ (see Supplementary Note 1 and Supplementary Table 1 for details). We operate all JPAs in the degenerate regime at the frequency *f*_0_ = 5.435 GHz with a pump frequency *f*_p_ = 2*f*_0_. The task of JPA 1 and JPA 2 is to generate propagating squeezed states which are incident to an entangling beam splitter. The resulting symmetric two-mode squeezed (TMS)^30,31^ states have a two-mode squeezing level^32^ of *S*_TMS_ = 7.1 dB and an entanglement strength characterized by the negativity criterion^30,33^ of *N* = 2.2. This number quantifies the strength of nonlocal correlations present between field quadratures of signals propagating along different beam splitter output paths. Additionally, the symmetric TMS states have negligible local squeezing within each path. In other words, the microwave signals propagating on the two paths locally look like thermal states with, nevertheless, strong entanglement between them (see Supplementary Note 2 and Supplementary Fig. 1 for details).

In the next step, we employ the symmetric propagating TMS states as a resource for remotely preparing the target squeezed microwave states. For this purpose, the TMS states are continuously distributed between two parties, Alice and Bob, who are separated by 35 of superconducting cable. Alice generates a feedforward signal carrying the classical information about her choice on what quantum state is to be remotely prepared at Bob’s side. Finally, Bob displaces his part of the resource state proportionally to the communicated signal by using a directional coupler with a fixed coupling of *β* ≃ −15 dB^34,35^. We experimentally implement the feedforward by operating JPA 3 as a phase-sensitive amplifier. Alice uses it to choose and strongly amplify a certain quadrature of the incoming TMS states. Note that, in contrast to the other JPAs, it does not matter whether the outgoing feedforward signal from JPA 3 is squeezed or not (Supplementary Note 5 and Supplementary Fig. 5). The essential classical information, as required for a successful RSP, is encoded in the large instantaneous amplitude of the phase-sensitively amplified field quadrature. For ideal RSP, the coupling *β* should be vanishingly small. However, since *β* needs to be approximately compensated by the degenerate gain of JPA 3, we are limited to a certain range of *β* values due to the noise and gain performance of JPA 3.

**Fig. 2:**
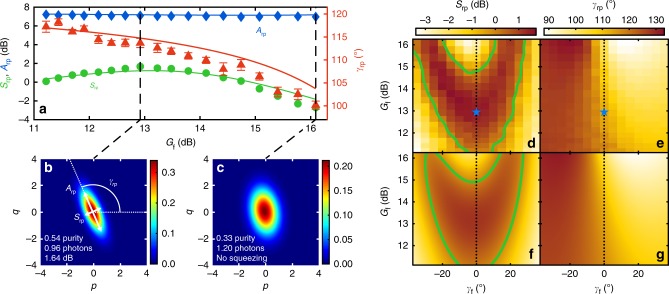
Tomography of remotely prepared states. **a** Squeezing level *S*_rp_ (circles), antisqueezing level *A*_rp_ (diamonds), and squeezing angle *γ*_rp_ (triangles) of remotely prepared states as a function of the JPA 3 degenerate gain *G*_f_ at fixed angle *γ*_f_ = 0°. The lines show a fit to the data. The error bars denote the standard error of the mean calculated from multiple repetitions of the protocol. If not shown, the error bars are smaller than the symbol size. **b**, **c** Reconstructed Wigner functions of the remotely prepared states for the optimal and one of the non-optimal JPA 3 gains as marked by the dashed lines in panel (**a**). **d**, **e** *S*_rp_ and *γ*_rp_ of the remotely prepared states as a function of the feedforward parameters. Panels (**f**), (**g**) show a joint fit of the three quantities (*S*_rp_, *A*_rp_, *γ*_rp_) to the corresponding data in panels (**d**), (**e**), respectively (see Supplementary Fig. 3 for the results for *A*_rp_). The green lines mark the threshold *S*_rp_ ≥ 0 dB for squeezing below the vacuum limit. The optimal point is marked by the blue star. The data and fit in panel (**a**) are marked by dotted lines in panels (**d**–**g**)

Figure 2a shows the experimental performance of the RSP scheme as a function of the JPA 3 degenerate gain *G*_f_ for a fixed JPA 3 amplification angle *γ*_f_ = 0°. The latter is defined as the deviation from the angle of the optimal working point at which we achieve the highest purity in the remotely prepared states. We fully characterize these states in terms of their squeezing level *S*_rp_, antisqueezing level *A*_rp_, and squeezing angle *γ*_rp_ (see “Methods”). We clearly observe squeezing up to *S*_rp_ = 1.6 ± 0.1 dB in the final states at the output of the displacer near the optimal JPA 3 gain *G*_f_ ≃ 13 dB (see Fig. 2b). *S*_rp_ decreases and the states even become non-squeezed upon deviation from the optimal JPA 3 gain as shown in Fig. 2c. The remotely prepared states can be encoded not only by varying *G*_f_ but also by changing *γ*_f_. The latter leads to a different quadrature in the resource TMS states being projected, and accordingly, to a different state being remotely prepared. The squeezing level and squeezing angle of the remotely prepared states obtained by sweeping both *G*_f_ and *γ*_f_ are shown in Fig. 2d, e. The results for the antisqueezing level *A*_rp_ can be found in Supplementary Fig. 3.

### Model and phase space of prepared states

Our experiment can be theoretically described by a model based on the input-output transformations for every component in the setup including transmission losses. In particular, we define *χ*_1_ as the total loss between JPA 1 (or JPA 2) and JPA 3, and *χ*_2_ as the total loss between JPA 1 (or JPA 2) and the directional coupler. This definition implies that all path losses between JPA 1 or JPA 2 and any component after the beam splitter are assumed to be equal (Supplementary Fig. 2). Additionally, we assume imperfect JPAs adding a certain amount of noise with mean thermal photon numbers *n*_*i*_ (*i* ∈ {1, 2, f}) to the JPA input signal. Here, *n*_f_ is the noise photon number of JPA 3. The RSP protocol is expected to work optimally for *G*_f_ = *τ*/(1 − *τ*) and *γ*_f_ = 0°, where *τ* = 1 − 10^*β*/10^ is the transmissivity of the directional coupler. At this optimal point and under the condition *S*_TMS_ ≥ 3 dB, we obtain the squeezed variance of the remotely prepared state,

1 $$\sigma _{\mathrm{s}}^2 = \frac{1}{4}\left[ {2(1 + 2n)e^{ - 2r}(1 - \chi )\tau + 2(\chi + n_{\mathrm{f}})\tau } \right].$$

In this simplified expression, we assume equal noise photon numbers *n*_1_ = *n*_2_ = *n* and squeezing factors *r*_1_ = *r*_2_ = *r* of JPA 1 and JPA 2 as well as equal losses *χ*_1_ = *χ*_2_ = *χ* (Supplementary Note 3). Equation (1) indicates that the prepared squeezing level *S*_rp_ at the optimal point is at least 3 dB below the squeezing level of the used resource. In order to correctly model the experiment, we additionally include a finite crosstalk between JPA 3 and the JPAs creating the TMS states as well as asymmetric losses *χ*_1_ ≠ *χ*_2_ in our data analysis. Figure 2f, g depicts a joint fit to the corresponding data. We observe a very good coincidence between the experimental results and our model for the following parameters (Supplementary Table 2): JPA 1, 2 squeezing levels *S* = 10.1 dB, *n* = 0.04, as well as *χ*_1_ = 0.22 and *χ*_2_ = 0.21 corresponding to losses of 1.1 dB and 1.0 dB, respectively. All values nicely agree with those obtained from independent JPA characterization measurements and loss estimations.

The quantum advantage of the RSP protocol consists in a smaller amount of classical information sent through the feedforward channel in order to prepare a desired state as compared to a purely classical protocol^8,36^. In the current experiment, this becomes evident by considering that only the amplified quadrature of the feedforward signal will affect the signal at Bob’s side due to the low coupling *β* ≪ 0 dB of the displacer. Consequently, we only send two real numbers while we are able to prepare different undisplaced mixed squeezed states which are fully described by three real numbers (*S*_rp_, *A*_rp_, *γ*_rp_).

**Fig. 3:**
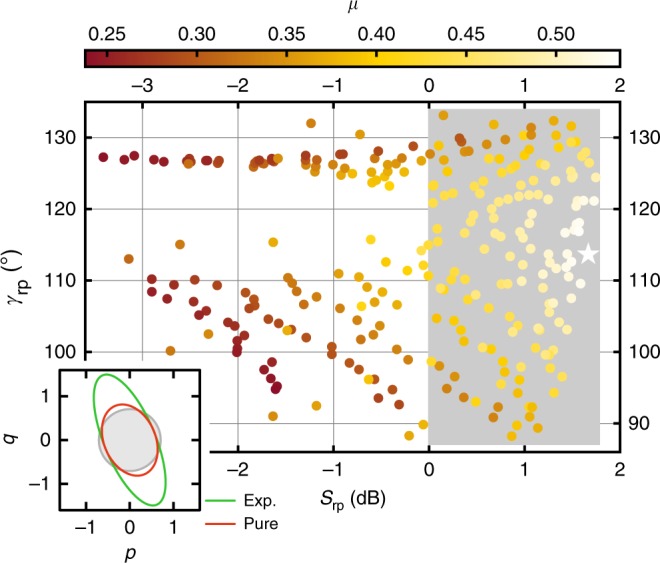
Purity of remotely prepared states in phase space. The phase space of the prepared states is spanned by *S*_rp_ and *γ*_rp_. The gray area marks squeezing below the vacuum limit. The color code indicates the purity *μ* of the remotely prepared states. The optimal point is marked by the white star. The inset shows the Wigner function 1/*e* contours of the experimental state at the optimal point with *μ* = 0.54 and a pure squeezed state with the same squeezing level. The vacuum 1/*e* contour is indicated in gray

The manifold of undisplaced Gaussian states we can prepare is intuitively understood by plotting the results from Fig. 2d, e in the phase space of the prepared squeezing level and angle, as it is shown in Fig. 3 (see Supplementary Note 4 and Supplementary Fig. 4 for details). The purity $\mu = 1/\left( {4\sqrt {{\mathrm{det}}\,\sigma } } \right)$, where *σ* is the covariance matrix of the remotely prepared state, incorporates the information about the antisqueezed quadrature and is a measure of how close the state is to a pure state. For application scenarios where pure states are desired, the performance of RSP can be best quantified by the purity. It vanishes for maximally mixed states and is unity for a pure state. We achieve the highest uncorrected purity *μ* = 0.54 ± 0.01 at the optimal point which is sufficient for many applications of squeezed states such as entanglement generation^33^, sideband cooling of optomechanical systems^37^, and quantum illumination^38^. Using appropriate modeling of our experiment, we are able to investigate the effect of imperfections in different parts of the protocol on the purity of the prepared state at the optimal point. If the resource TMS state is pure and distributed ideally to Alice and Bob, the model predicts an improved purity of 0.60 for otherwise unchanged parameters. Alternatively, ideal operations of Alice and Bob (noiseless JPA 3 combined with no losses on Alice’s and Bob’s sides) with a realistic entangled resource would yield a purity of 0.62. Overall, the observed purity is limited by the added noise of the JPAs and the losses in the setup. Upon reducing the JPA noise photon numbers by one order of magnitude as well as the total losses to *χ*_1_ = *χ*_2_ = 0.05 (0.2 dB), we expect an optimized purity *μ*_opt_ = 0.80 for the prepared state at the optimal point. The reduction of losses can be achieved by using a superconducting hybrid ring, optimized cable connectors, and improved circulators. In this context, one should remember that our protocol allows for the preparation of continuous-variable squeezed states with a degree of squeezing *S*_rp_ that is fundamentally related to the initial two-mode squeezing of the resource state. In the current implementation, even for a fixed resource TMS state, *S*_rp_ and *γ*_rp_ can be changed at the expense of a reduced purity *μ*. By adding a phase shifter^39^ on her side, Alice could prepare squeezed states with arbitrary *γ*_rp_ while keeping *S*_rp_ and *μ* constant.

### Quantum one-time pad

**Fig. 4:**
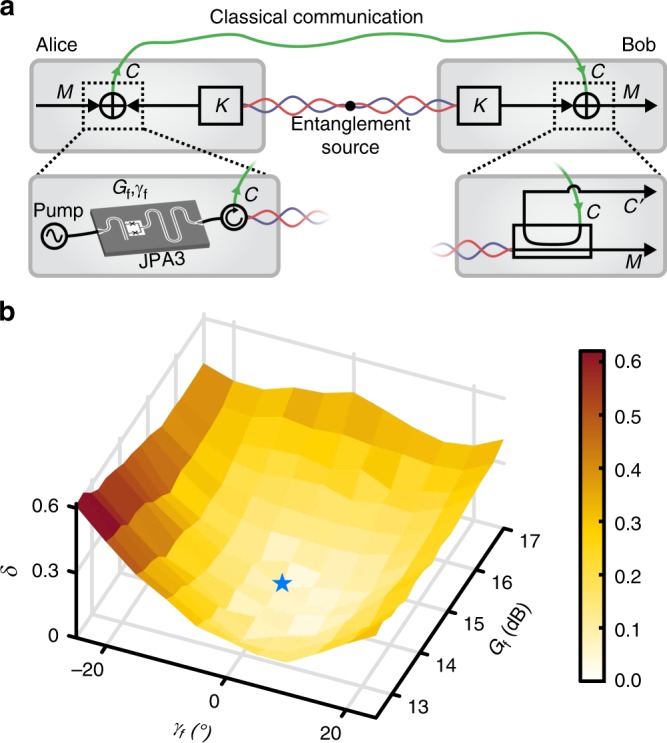
Quantum one-time pad. **a** Scheme for the interpretation of the RSP setup in terms of the one-time pad. **b** Entropy difference *δ* = *H*(*M*) − *H*(*M*|*C*′) as a function of the feedforward gain *G*_f_ and angle *γ*_f_. The optimal point is marked by the blue star. It is slightly shifted with respect to the previous measurements due to a modified cryogenic setup

Finally, we relate our experimental RSP scheme to the cryptographic protocol known as the one-time pad by extending the latter to the quantum regime^8,40^. Here, Alice securely sends a quantum state *M* to Bob over an insecure channel. We identify the transmitted message *M* as the remotely prepared state on Bob’s side and the openly communicated cipher *C* as the feedforward signal (see Fig. 4a). The entangled TMS states provide the random key *K* in the form of quantum fluctuations to both parties. Note that *K* is essential for the one-time pad since it is used by Alice and Bob to encode and decode *M*. For secure communication, *K* needs to be a uniform random variable, such that an eavesdropper with knowledge about *C* does not gain any information about *M*^41^. Formally, we can write

2 $$H(M) - H(M|C) = 0{\kern 1pt} ,$$

where *H*(*M*) is the von Neumann entropy of the remotely prepared state and *H*(*M*|*C*) = *H*(*M*, *C*) − *H*(*C*) is the conditional entropy of *M* given the feedforward signal (Supplementary Note 6). We experimentally investigate the quantum one-time pad by measuring the prepared states as a function of the JPA 3 parameters, while additionally detecting the signal *C*′ from the second directional coupler output. We compute *δ* = *H*(*M*) − *H*(*M*|*C*′) to verify Eq. (2) under the reasonable approximation *C*′ ≈ *C* (due to *τ* ≃ 1) using state tomography. In Fig. 4b, we observe a decrease in *δ* when moving towards the optimal point where the smallest value *δ* = 0.06 ± 0.04 is reached and the entropy of the prepared state is *H*(*M*) = 0.80 ± 0.02. The observation $\delta \ll H(M)$ indicates that the remote preparation of a quantum state on Bob’s side is close to perfect security when approaching the optimal point. At the same time, there is a trade-off between the security and range of prepared states. As previously mentioned, a straightforward extension of the optimal working range of RSP can be realized by adding a phase shifter on Alice’s side. Then, all optimally prepared states would fulfill the security criterion *H*(*M*) = *H*(*M*|*C*) for arbitrary squeezing angles while the accessible squeezing level and purity stay unaffected. It is important to note that a potential eavesdropper cannot obtain any information about *M* by listening exclusively to the TMS resource state since the encoding of *M* happens later in the protocol. Furthermore, if the eavesdropper completely disentangles the TMS state by its actions, RSP yields no squeezing in the final state which could be exploited to detect the eavesdropper’s presence.

## Discussion

To conclude, we have successfully implemented a deterministic RSP protocol over a distance of 35 cm in the microwave regime with continuous variables and explored the influence of different parameters on the remotely prepared states. We have remotely prepared squeezed states with a squeezing level of up to 1.6 dB below the vacuum limit. In our specific RSP implementation, Alice can control the squeezing level and, to some extent, the squeezing angle of the remotely prepared state at the expense of a reduced purity. Additionally, we demonstrate that the protocol can be interpreted as a secure one-time pad near the optimal point. The operational range of both the RSP and quantum one-time pad protocols can be extended to any angle *γ*_rp_ by an additional phase shifter on Alice’s side. The demonstrated protocol opens a way to a multitude of intriguing experiments with quantum microwaves such as probing the Holevo bound limits^42^, studying the role of quantum discord in quantum communication protocols^43^, exploring hybrid continuous-discrete schemes of quantum information processing^44^, and implementing quantum illumination protocols^38^. Squeezing operations can further be exploited for the preparation of Gottesman-Kitaev-Preskill (GKP) states for continuous-variable quantum error correction^45,46^. Our experiment proves that prototypical local quantum networks using continuous-variable quantum microwaves are within experimental reach^47^.

## Methods

JPA 1 and JPA 2 perform a squeezing operation $\hat S(\xi )|0\rangle$, where $\hat S(\xi ) = {\mathrm{exp}}\left( {\frac{1}{2}\xi ^ \ast \hat a^2 - \frac{1}{2}\xi (\hat a^\dagger )^2} \right)$ is the squeezing operator, $\hat a^\dagger = \hat q - i\hat p$ and $\hat a = \hat q + i\hat p$ are the creation and annihilation operators with $\left[ {\hat a,\hat a^\dagger } \right] = 1$ of the *f*_0_ mode with quadratures $\hat q$ and $\hat p$, and *ξ* = *re*^*iϕ*^ is the complex squeezing amplitude. Here, the phase *ϕ* = − 2*γ* determines the squeezing angle *γ* between the antisqueezed quadrature and the *p*-axis in the phase space, while the squeezing factor *r* parameterizes the amount of squeezing. We define the degree of squeezing in decibels as $S = - 10{\kern 1pt} \,\log _{10}\left[ {\sigma _{\mathrm{s}}^2/0.25} \right]$, where $\sigma _{\mathrm{s}}^2$ is the variance of the squeezed quadrature and the vacuum variance is 0.25. Positive values of *S* indicate squeezing below the vacuum level. The antisqueezing level is defined as $A = 10\,{\mathrm{log}}_{10}\left[ {\sigma _{\mathrm{a}}^2/0.25} \right]$, where $\sigma _{\mathrm{a}}^2$ is the variance of the antisqueezed quadrature. We generate symmetric TMS states at the outputs of the hybrid ring by pumping JPA 1 and JPA 2 with strong quasi-continuous microwave drives, so that they produce squeezed states with the same squeezing level and orthogonal squeezing angles *γ*_2_ = *γ*_1_ + *π*/2. These angles are stabilized by controlling the respective pump phases employing a phase-locked loop^30,34^. The stability of these TMS states in terms of two-mode squeezing and symmetry is of paramount importance in our experiments. Only by utilizing the nonclassical correlations between Alice and Bob, it is possible to demonstrate the successful RSP protocol. In order to reconstruct the quantum states in the experiment, we employ a well-tested reference state tomography based on statistical moments of the detected field quadratures^32,33^.

## Supplementary information

Supplementary Information

## Data Availability

The data that support the findings of this study are available from the corresponding authors upon reasonable request.
