# Supplementary material for: Secure quantum remote state preparation of squeezed microwave states
Source: Nat Commun. 2019 Jun 13;10:2604. doi: 10.1038/s41467-019-10727-7 (PMC6565634; doi:10.1038/s41467-019-10727-7)
Supplement: Supplementary file 1 — Supplementary Information [file 41467_2019_10727_MOESM1_ESM.pdf]

Supplementary Information for:

Secure quantum remote state preparation of squeezed microwave states

Pogorzalek *et al.*

SUPPLEMENTARY NOTE 1: JOSEPHSON PARAMETRIC AMPLIFIERS

The Josephson parametric amplifiers (JPA) used in this work consist of a quarter-wavelength superconducting microwave resonator in a coplanar waveguide geometry which is short-circuited to the ground plane via a direct current superconducting quantum interference device (dc-SQUID)[1,2]. The JPAs were designed and fabricated at NEC Smart Energy Research Laboratories, Japan and RIKEN, Japan. The resonator and pump line are patterned into a 50 nm thick Nb film which has been deposited by magnetron sputtering onto 300 μm thick silicon substrates covered by a thermal oxide. The dc-SQUID is fabricated using an aluminum shadow evaporation technique. The flux-tunable resonant frequency f_0 of the JPA can be tuned by an external magnetic flux applied to the dc-SQUID loop via an external coil or via an on-chip antenna acting as the pump line. In order to squeeze incoming vacuum fluctuations or perform phase-sensitive amplification of the mode f_0, we apply a strong coherent pump tone at frequency $f_\mathrm{p} = 2f_0$ to the pump line. The squeezing strength (degenerate gain) and squeezing angle (amplified quadrature) are controlled by the pump amplitude and pump phase, respectively, when the JPA is operated as squeezer (degenerate amplifier). For each JPA, a commercial cryogenic circulator is used to separate the incoming from the outgoing signal (see Supplementary Figure 1). In order to pre-characterize the JPAs and find a suitable working frequency f_0 for all JPAs, we perform spectroscopic measurements[2]. The extracted parameters are summarized in Supplementary Table 1.

Supplementary Table 1: JPA Parameters extracted by fitting the dependence of the resonant frequency f_0 of the JPAs on the applied magnetic flux[2]. Here, I_c and $E_\mathrm{J} = I_c\Phi_0/2\pi$ are the critical current and coupling energy of a single Josephson junction, respectively, L_loop and $\beta_\mathrm{L} = 2L_\mathrm{loop}I_c/\Phi_0$ are the loop inductance and screening parameter of the dc-SQUID, respectively, and f_r is the resonant frequency of the bare resonator. The Josephson junctions of the dc-SQUID are assumed to be identical. The external quality factors Q_ext and internal quality factors Q_int are obtained from independent fits of the JPA spectral linewidths[2]. The parameters of JPA 3 are similar to the ones of JPA 1 and JPA 2.

Sample	I_c (μA)	β_L	L_loop (pH)	$f_\mathrm{r}/2\pi$ (GHz)	E_J/h (THz)	Q_ext	Q_int
JPA 1	2.45	0.09	35.8	5.808	1.22	300–360	>30000
JPA 2	2.41	0.10	40.7	5.838	1.20	240–260	>30000

SUPPLEMENTARY NOTE 2: EXPERIMENTAL SETUP

The experimental room temperature and cryogenic setup is shown in Supplementary Figure 1. The digitizer card and the microwave pump sources for each JPA are pulsed with a data timing generator (DTG). JPA 1 and JPA 2 are both temperature stabilized at 50 mK in order to ensure a stable JPA operation and produce squeezed states with orthogonal squeezing angles. The two squeezed states are superimposed by a cryogenic hybrid ring (50:50 beam splitter) in order to produce path-entangled two-mode squeezed (TMS) states at the outputs of the hybrid ring. By operating JPA 1 and JPA 2 at the same squeezing level, we are able to produce symmetric TMS states with local statistics of a thermal state. The entanglement strength of the TMS states is characterized by the two-mode squeezing level of $S_\mathrm{TMS} = 7.1$ dB and the negativity criterion of $N = 2.2$. Additionally, a lower bound on the entanglement of formation, $E_\mathrm{F} = 1.2$, can be calculated for our experimental TMS states[3]. One output path of the beam splitter is connected to JPA 3 which is operated as a phase-sensitive amplifier. The JPA 3 output signal is then either detected or sent to a directional coupler which couples to the other hybrid ring output. The first amplification stage of a high-electron-mobility transistor (HEMT) is followed by additional rf-amplifiers which are temperature stabilized with a Peltier cooler. We use a vector network analyzer for the characterization of the JPAs and a heterodyne detection setup for the tomographic measurements.
The heterodyne detection setup and data processing is similar to those described in Supplementary References 4,5 where the signal is roughly filtered around the working frequency and down-converted to 11 MHz by image rejection mixers. The signal is then digitized with analog-to-digital (ADC) converters on an Acqiris DC440 card. After sending the digitized data to a computer, digital data processing is performed where digital down-conversion (DDC) and finite-impulse response (FIR) filtering with a full bandwidth of 430 kHz is applied. Finally, all correlation quadrature moments $\langle I_1^n I_2^m Q_1^k Q_2^l \rangle$ with $n+m+k+l \leq 4$ for $n, m, k, l \in \mathbb{N}$ are calculated and averaged. The data within a single averaging cycle consists of 4×10^8 averaged sample points per part of the pulse and is used to perform a reference state reconstruction for each pulse in order to obtain the signal moments $\langle (\hat{a}^\dagger)^n \hat{a}^m \rangle$ with $n+m \leq 4$. During each measurement cycle, the moments of JPAs 1-3 are used to calculate the squeezing angles γ_i^exp for each JPA "on the fly" in order to obtain the angle correction $\delta\gamma_i = \gamma_i^\mathrm{ext} - \gamma_i^\mathrm{target}$ which is used to adjust the phase of the microwave pump tone by $2\delta\gamma_i$. Finally, the described averaging cycle is repeated 10 times. The vector network analyzer, DTG,

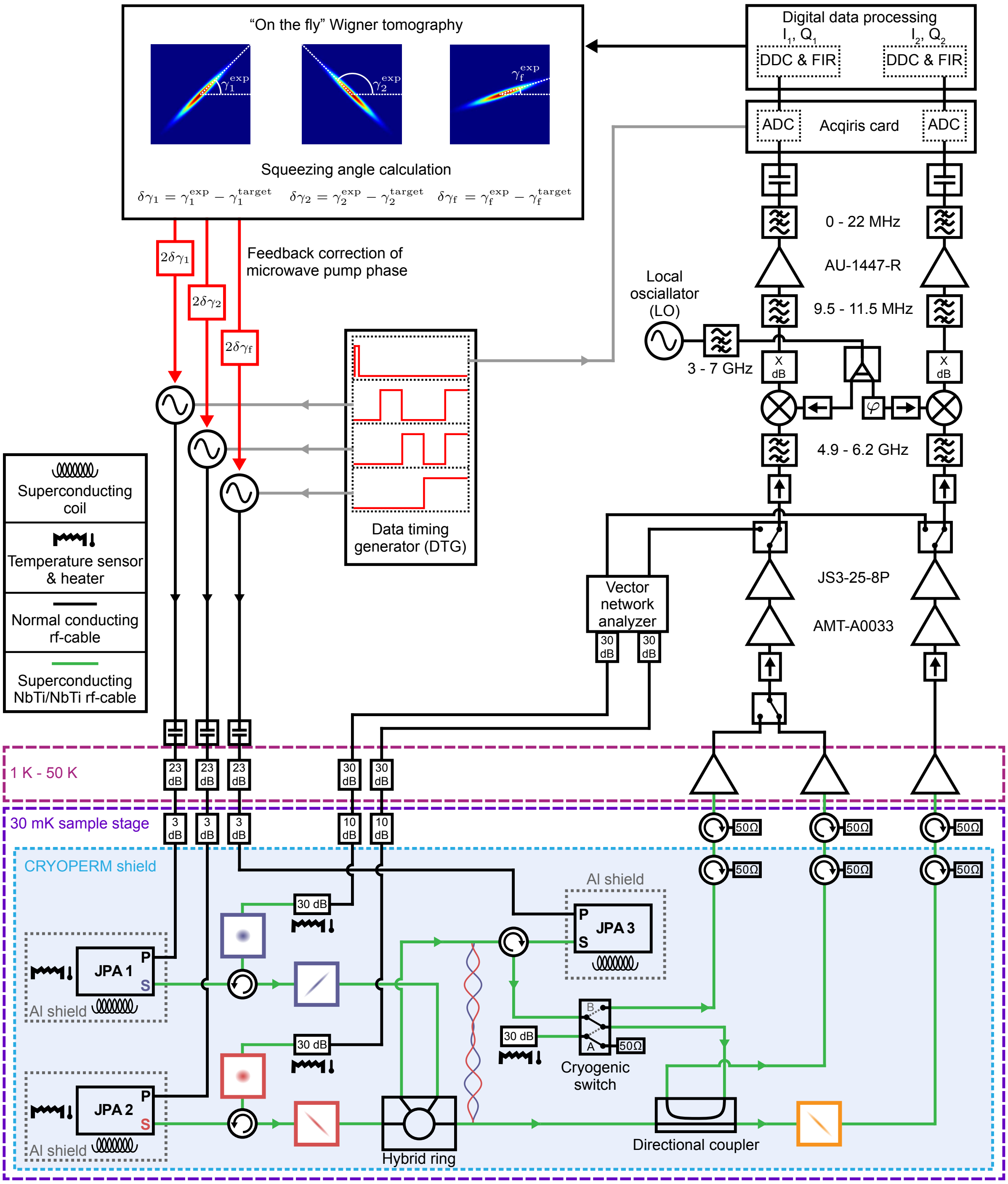

Supplementary Figure 1: Experimental scheme for the measurements. The JS3-25-8P rf-amplifiers are removed for the measurements concerning the quantum one-time pad. The RSP and quantum one-time pad measurements are performed with the cryogenic switch in position A. The intertwined lines between the outputs of the hybrid ring symbolize the entanglement. JPA 3 and the directional coupler are separated by 35 cm of superconducting cable.

Acqiris card and local oscillator are synchronized to a 10 MHz rubidium frequency standard. The pump microwave sources are daisy chained to the local oscillator with a 1 GHz reference signal.
The experimental states are reconstructed under the assumption that the states are Gaussian, and thus fully described by their signal moments up to the second order. In order to check for the Gaussianity of the states, we verify that the cumulants of third and fourth order are vanishingly small, as expected for Gaussian states[6]. The cumulants

$\langle\langle(\hat{a}^\dagger)^n\hat{a}^m\rangle\rangle$ are calculated from the signal moments $\langle(\hat{a}^\dagger)^n\hat{a}^m\rangle$ according to

$$\langle\langle(\hat{a}^\dagger)^n\hat{a}^m\rangle\rangle = \partial_x^n\partial_y^m \ln\sum_{\alpha,\beta}\frac{\langle(\hat{a}^\dagger)^\alpha\hat{a}^\beta\rangle x^\alpha y^\beta}{\alpha!\beta!}\bigg|_{x=y=0}\,, \tag{1}$$

where ∂_x^n is the n-th partial derivative with respect to x and ln is the natural logarithm[7,8].

SUPPLEMENTARY NOTE 3: THEORETICAL MODELING AND FITTING PROCEDURE

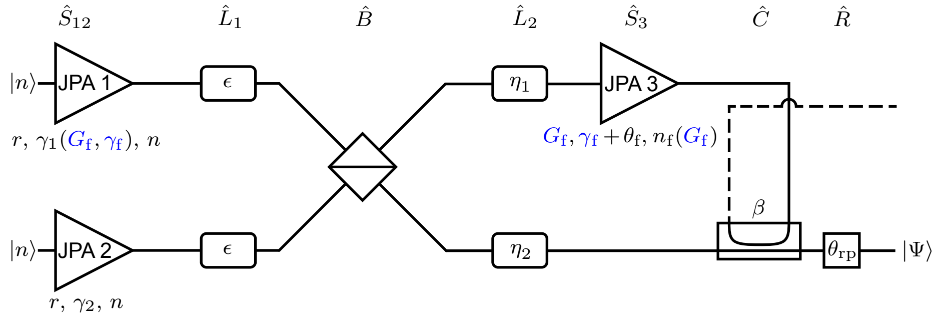

Supplementary Figure 2: Scheme for theoretical description of the RSP setup with used parameters. The experimentally varied feedforward gain G_f and angle γ_f are marked in blue. Due to a carefully designed symmetric implementation in the experiment, the losses $\epsilon_1 = \epsilon_2 = \epsilon$ in both paths before the beam splitter are assumed to be equal and include the insertion loss of the beam splitter. The angles γ_1, γ_2 and γ_f are used in units of radians for all equations.

In order to theoretically describe the remote state preparation (RSP) setup, we use an input-output model for each component as shown in Supplementary Figure 2. JPA 1 and JPA 2 are modeled as squeezers with the same squeezing parameter $r_1 = r_2 = r$ but different squeezing angles γ_1 and γ_2 in order to produce symmetric TMS states after the beam splitter. In the experiment, the pump of JPA 3 leaks through to JPA 1 and JPA 2 which results in a finite crosstalk between JPA 3 and the other two JPAs. Since experimentally the crosstalk to JPA 1 dominates, we approximate the effect of the crosstalk by a linear dependence of γ_1 on the gain G_f and angle γ_f of JPA 3

$$\gamma_1 = \gamma_1^{(0)} + \kappa G_\mathrm{f} + \lambda\gamma_\mathrm{f}\,, \tag{2}$$

where $\gamma_1^{(0)}$ is the unperturbed squeezing angle of JPA 1. This approximation is consistent with independent measurements of the crosstalk. The squeezing operator $\hat{S}_{12}$ for JPA 1 and JPA 2 acting on the annihilation operators $\hat{a}_i$ of path 1 (Alice) and path 2 (Bob) is given by[9]

$$\hat{S}_{12}^\dagger\begin{pmatrix}\hat{a}_1\\ \hat{a}_2\end{pmatrix}\hat{S}_{12} = \begin{pmatrix}\hat{a}_1\cosh r - \hat{a}_1^\dagger e^{-2i\gamma_1}\sinh r\\ \hat{a}_2\cosh r - \hat{a}_2^\dagger e^{-2i\gamma_2}\sinh r\end{pmatrix}. \tag{3}$$

The added noise of JPA 1 and JPA 2 is taken into account by an effective thermal state with a noise photon number $n_1 = n_2 = n$ incident to the JPAs. In order to describe the action of JPA 3, we assume that classical noise is added to the JPA input signal followed by ideal phase-sensitive amplification

$$\hat{S}_3^\dagger\begin{pmatrix}\hat{a}_1\\ \hat{a}_2\end{pmatrix}\hat{S}_3 = \begin{pmatrix}(\hat{a}_1+\zeta)\cosh r_\mathrm{f} - \left(\hat{a}_1^\dagger+\zeta^*\right)e^{-2i(\gamma_\mathrm{f}+\theta_\mathrm{f})}\sinh r_\mathrm{f}\\ \hat{a}_2\end{pmatrix}, \tag{4}$$

where G_f is related to r_f as $G_\mathrm{f} = e^{2r_\mathrm{f}}$ and θ_f is the theoretically optimal JPA 3 amplification angle. The classical noise is described by the complex Gaussian random variable ζ with $\langle\zeta\rangle = 0$, $\langle\zeta\zeta^*\rangle = n_\mathrm{f}$ and $\langle\mathrm{Re}(\zeta)^2\rangle = \langle\mathrm{Im}(\zeta)^2\rangle = n_\mathrm{f}/2$, where n_f is the effective thermal noise photon number. In general, the JPA noise is gain dependent which we take into account by a linear dependence on G_f for JPA 3. For that, we use $n_\mathrm{f} = n_\mathrm{f}' G_\mathrm{f}$, where n_f' is a proportionality constant. Losses ϵ and η_i of the microwave components are modeled with a beam splitter[10]

$$\hat{L}_1^\dagger\begin{pmatrix}\hat{a}_1\\ \hat{a}_2\end{pmatrix}\hat{L}_1 = \begin{pmatrix}\sqrt{1-\epsilon}\,\hat{a}_1 + \sqrt{\epsilon}\,\hat{v}_1\\ \sqrt{1-\epsilon}\,\hat{a}_2 + \sqrt{\epsilon}\,\hat{v}_2\end{pmatrix}, \tag{5}$$

$$\hat{L}_2^\dagger \begin{pmatrix} \hat{a}_1 \\ \hat{a}_2 \end{pmatrix} \hat{L}_2 = \begin{pmatrix} \sqrt{1-\eta_1}\hat{a}_1 + \sqrt{\eta_1}\hat{v}_1 \\ \sqrt{1-\eta_2}\hat{a}_2 + \sqrt{\eta_2}\hat{v}_2 \end{pmatrix} , \tag{6}$$

where $\hat{v}_i$ is the operator describing the environment for path i. The environment can be safely approximated to be in the vacuum state due to the low temperature of the lossy components in the experiment.
The hybrid ring is described by a 50:50 beam splitter[11]

$$\hat{B}^\dagger \begin{pmatrix} \hat{a}_1 \\ \hat{a}_2 \end{pmatrix} \hat{B} = \frac{1}{\sqrt{2}} \begin{pmatrix} \hat{a}_1 + \hat{a}_2 \\ -\hat{a}_1 + \hat{a}_2 \end{pmatrix} . \tag{7}$$

The displacement on Bob's side is implemented with a directional coupler and is described as an asymmetric beam splitter[12]

$$\hat{C}^\dagger \begin{pmatrix} \hat{a}_1 \\ \hat{a}_2 \end{pmatrix} \hat{C} = \begin{pmatrix} \sqrt{\tau}\hat{a}_1 + \sqrt{1-\tau}\hat{a}_2 \\ -\sqrt{1-\tau}\hat{a}_1 + \sqrt{\tau}\hat{a}_2 \end{pmatrix} , \tag{8}$$

where $\tau = 1 - 10^{\beta/10}$ is the transmissivity and β is the coupling in decibel.
In order to describe the realistic setup, we need to take the electrical length of the different components into account. The total electrical lengths as well as different path lengths after the beam splitter are compensated with a rotation $\hat{R}$ by the angle θ_{rp} of the final remotely prepared state on Bob's side

$$\hat{R}^\dagger \begin{pmatrix} \hat{a}_1 \\ \hat{a}_2 \end{pmatrix} \hat{R} = \begin{pmatrix} \hat{a}_1 \\ \hat{a}_2 e^{-i\theta_{\mathrm{rp}}} \end{pmatrix} . \tag{9}$$

With the operator definitions in Supplementary Equations (3)–(9), we can write the overall RSP protocol as

$$|\Psi\rangle = \hat{R}\,\hat{C}\,\hat{S}_3\,\hat{L}_2\,\hat{B}\,\hat{L}_1\,\hat{S}_{12}|n,n\rangle , \tag{10}$$

where n is the noise photon number of JPA 1 and JPA 2, and $|\Psi\rangle$ is the final state on both paths. The moment matrices for both paths of the final state are calculated as

$$\begin{pmatrix} \langle(\hat{b}^\dagger)^n\hat{b}^m\rangle_1 \\ \langle(\hat{b}^\dagger)^n\hat{b}^m\rangle_2 \end{pmatrix} = \langle\Psi| \begin{pmatrix} (\hat{a}^\dagger)_1^n\hat{a}_1^m \\ (\hat{a}^\dagger)_2^n\hat{a}_2^m \end{pmatrix} |\Psi\rangle , \tag{11}$$

where $\langle(\hat{b}^\dagger)^n\hat{b}^m\rangle_1$ are the moments of the second directional coupler output signal and $\langle(\hat{b}^\dagger)^n\hat{b}^m\rangle_2$ are the moments of the remotely prepared state. With the definition of the quadratures $\hat{q} = (\hat{b}+\hat{b}^\dagger)/2$ and $\hat{p} = (\hat{b}-\hat{b}^\dagger)/2i$, the moments $\langle(\hat{b}^\dagger)^n\hat{b}^m\rangle_2$ are used to calculate the squeezing angle γ_{rp}, squeezed variance σ_{s}^2 and antisqueezed variance σ_{a}^2 of the remotely prepared state as

$$\gamma_{\mathrm{rp}} = -\frac{1}{2}\arg\left(-\langle\hat{b}^2\rangle_2\right) , \tag{12}$$

$$\sigma_{\mathrm{s}}^2 = \frac{1}{4}\left(\langle\hat{b}^2\rangle_2 e^{2i\gamma_{\mathrm{rp}}} + \langle(\hat{b}^\dagger)^2\rangle_2 e^{-2i\gamma_{\mathrm{rp}}} + 2\langle\hat{b}^\dagger\hat{b}\rangle_2 + 1\right) , \tag{13}$$

$$\sigma_{\mathrm{a}}^2 = \frac{1}{4}\left(-\langle\hat{b}^2\rangle_2 e^{2i\gamma_{\mathrm{rp}}} - \langle(\hat{b}^\dagger)^2\rangle_2 e^{-2i\gamma_{\mathrm{rp}}} + 2\langle\hat{b}^\dagger\hat{b}\rangle_2 + 1\right) , \tag{14}$$

where $\arg(\cdot)$ is the argument of a complex number and the first order moments are taken to be zero. These quantities are then fitted simultaneously to the corresponding quantities of the experimental remotely prepared states (see Supplementary Figure 3 for antisqueezed variance). We are able to describe the RSP protocol presented in the main article with the parameters shown in Supplementary Table 2. We emphasize that the bare model only requires three fitting parameters (n, r, n_{f}') in order to obtain a good fit when estimating the remaining parameters from independent measurements. Including β, θ_{f}, θ_{rp}, and the crosstalk parameters ($\gamma_1^{(0)}$, κ, λ) as fitting parameters, only slightly improves the quantitative agreement between the experiment and the theory.
In order to derive equation (1) in the main article, we choose $\gamma_1 = \gamma_{\mathrm{f}} = 0°$, $\gamma_2 = \theta_{\mathrm{f}} = 90°$, same losses after the beam splitter ($\eta_1 = \eta_2$), neglect the effect of the electrical path lengths ($\theta_{\mathrm{rp}} = 0$), and define the total loss $\chi_1 = \chi_2 = \chi$, where $\chi_1 = 1-(1-\epsilon)(1-\eta_1) = \epsilon + \eta_1 - \epsilon\eta_1$ and $\chi_2 = 1-(1-\epsilon)(1-\eta_2) = \epsilon + \eta_2 - \epsilon\eta_2$. Furthermore, we do not consider the experimental crosstalk ($\kappa = \lambda = 0$). The protocol works optimally for fixed resources if a state with the highest purity is

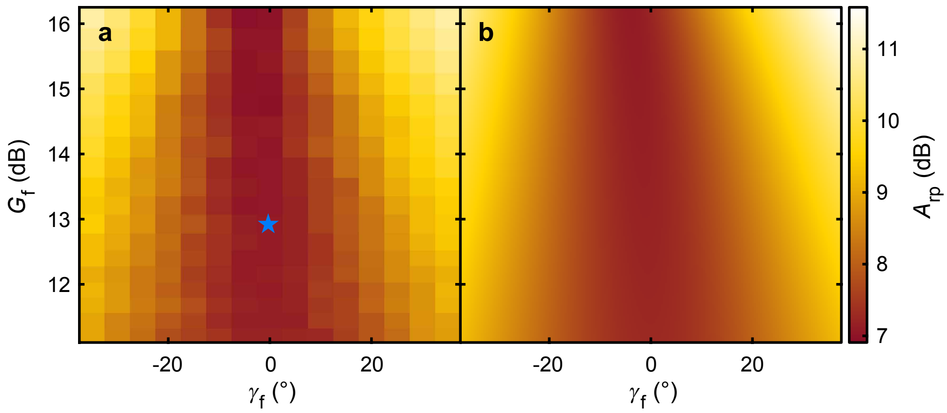

Supplementary Figure 3: **a**,**b** Antisqueezed variance $A_{\rm rp}$ of the remotely prepared states as a function of the feedforward parameters for experiment and fit. The optimal point is marked by the blue star.

Supplementary Table 2: Model parameters used to theoretically describe the RSP protocol in the main article. The losses ϵ, η_1 and η_2 are estimated from the individual loss of the components. γ_2 is fixed to the experimentally chosen squeezing angle of JPA 2.

n	r	$\gamma_1^{(0)}$ (°)	γ_2 (°)	$n'_{\rm f}$	β (dB)	ϵ	η_1	η_2	$\theta_{\rm f}$ (°)	$\theta_{\rm rp}$ (°)	κ (°)	λ
0.04	1.20	49.6	135.0	0.0059	−14.6	0.15	0.08	0.07	136.5	68.5	−0.17	0.02

remotely prepared. In the limit of high JPA 1 and JPA 2 squeezing, $r \gg 1$, we reach this optimal point for $G_{\rm f} = \tau/(1-\tau)$ and obtain for the optimally remotely prepared state by using Supplementary Equations (12)-(14)

$$\tilde{\gamma}_{\rm rp} = \gamma_1 \,, \tag{15}$$

$$\tilde{\sigma}_{\rm s}^2 = \frac{1}{4}\left[2(1+2n)e^{-2r}(1-\chi)\tau + 2(\chi + n_{\rm f})\tau\right] \,, \tag{16}$$

$$\tilde{\sigma}_{\rm a}^2 = \frac{(1+2n)(1-\chi)\left[e^{2r} + e^{-2r}(1-2\tau)^2\right]}{8\tau} + \frac{2n_{\rm f}(1-\tau)^2 + \left(1-2\tau+2\tau^2\right)\chi}{4\tau} \,. \tag{17}$$

In general, the optimal JPA 3 gain depends on r in a nontrivial manner and converges to $G_{\rm f} = \tau/(1-\tau)$ for $r \to \infty$. However, the latter expression offers a good approximation to the optimal JPA 3 gain even for $r \approx 1$ since the deviation of $\tilde{\gamma}_{\rm rp}$, $\tilde{\sigma}_{\rm s}^2$, and $\tilde{\sigma}_{\rm a}^2$ between the optimal JPA 3 gain and $G_{\rm f} = \tau/(1-\tau)$ is below 1% for the parameters in Supplementary Table 2.

SUPPLEMENTARY NOTE 4: PHASE SPACE OF PREPARABLE STATES

The model described in the previous section allows us to theoretically investigate the phase space of the preparable states of our RSP protocol in the phase space spanned by ($S_{\rm rp}$, $\gamma_{\rm rp}$) and ($S_{\rm rp}$, $A_{\rm rp}$). For this purpose, we use the parameters from Supplementary Table 2 and calculate the contour around the remotely prepared states for the experimental range of JPA 3 gain $G_{\rm f}$ and amplification angle $\gamma_{\rm f}$ (green contour in Supplementary Figure 4a,b). Alternatively, we use an iterative method to calculate the maximum error contour (blue contour in Supplementary Figure 4a,b). Here, we randomly select a value from the 95% confidence intervals of each fitting parameter and calculate the resulting contour. If the current contour lies partly or fully outside the maximum error contour, the latter is expanded so that it includes the current contour. The process is repeated until the change of the area of the maximum error contour is negligible between iterations.

We observe that the direct contour does not include all experimentally prepared states but shows a good qualitative agreement. The maximum error contour includes all measured remotely prepared states. We note that all remotely prepared states inside the contour can be continuously prepared. However, the position in the phase space does not uniformly depend on $G_{\rm f}$ and $\gamma_{\rm f}$. Since we select a finite and uniform step size of $G_{\rm f}$ and $\gamma_{\rm f}$ in the experiment, the measured remotely prepared states do not uniformly occupy the phase space.

In Supplementary Figure 4c,d, we show the expected contours of the accessible prepared states upon reducing the JPA noise photon numbers by one order of magnitude and the different losses (ϵ, η_1 and η_2) to 0.1 dB each. The

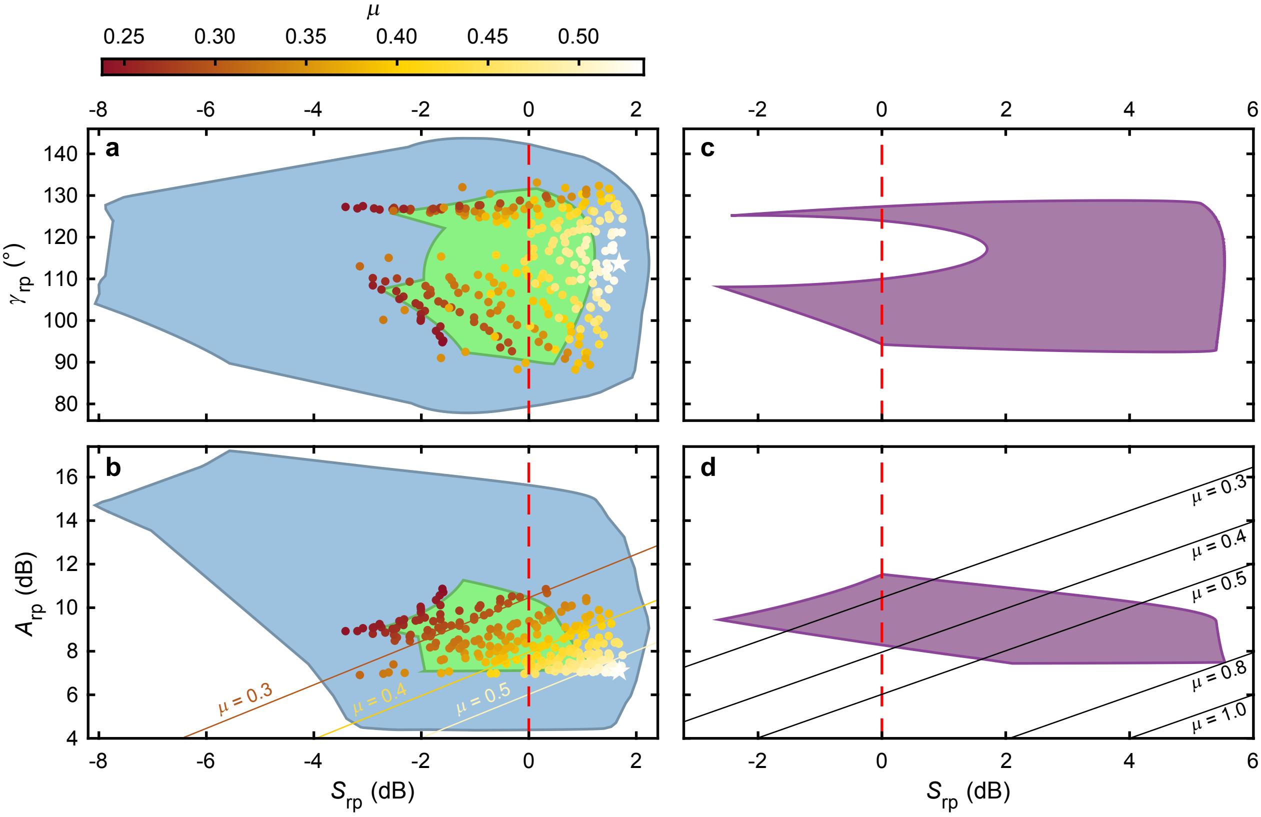

Supplementary Figure 4: **a**,**b** Phase space of experimental remotely prepared states spanned by ($S_{\rm rp}$, $\gamma_{\rm rp}$) and ($S_{\rm rp}$, $A_{\rm rp}$). The green and blue shaded area indicate the direct contour from the fit and maximum error contour, respectively. The color code indicates the purity μ of the remotely prepared states. The optimal point is marked by a white star. **c**,**d** Expected theoretical contours of preparable states for an improved setup in the phase space spanned by ($S_{\rm rp}$, $\gamma_{\rm rp}$) and ($S_{\rm rp}$, $A_{\rm rp}$). Here, we reduce the JPA noise photon numbers by one order of magnitude and the losses to $\epsilon = \eta_1 = \eta_2 = 0.023$ with otherwise unchanged parameters from Supplementary Table 2. The red dashed lines mark the threshold for squeezing below the vacuum limit. The solid lines mark states with constant purity.

range of preparable $A_{\rm rp}$ and $\gamma_{\rm rp}$ is only slightly affected by the optimized parameters while the squeezing level $S_{\rm rp}$ is significantly increased.

SUPPLEMENTARY NOTE 5: FEEDFORWARD SIGNAL

The feedfoward signal is characterized by toggling the cryogenic switch into position B (see Supplementary Figure 1) and measure both the signal from JPA 3 (Alice's side) as well as the signal on Bob's side while all JPAs are pumped. The squeezing level of JPA 1 and JPA 2 for these measurements is $S = 7.3$ dB which results in an entangled state with a negativity kernel[6,13] $N_{\rm k} = 1.8$ after the beam splitter. The JPA 3 amplification angle is fixed to the optimal angle $\gamma_{\rm f} = 0°$. As shown in Supplementary Figure 5, we observe no entanglement ($N_{\rm k} \leq 0$) between Alice and Bob after the local amplification for $G_{\rm f} \geq 11$ dB. The feedforward signal is squeezed below the vacuum for low $G_{\rm f}$ and becomes non-squeezed above $G_{\rm f} \simeq 13$ dB. Our theory model and experimental evidence show that the deamplified, and possibly squeezed, quadrature has a negligible effect on the prepared state. This can be understood by considering that the feedforward signal is only weakly coupled to Bob's part of the entangled state by the directional coupler. Therefore, only the strongly amplified quadrature in the feedforward signal will affect the prepared state on Bob's side.
We consider the feedforward signal as classical if it has a positive Wigner function, is not squeezed below the vacuum and is not entangled with the signal on Bob's side. Therefore, all feedforward signals with $G_{\rm f} \geq 13$ dB are classical. For $G_{\rm f} < 13$ dB, the information about the to-be-prepared state in the feedforward signal can be described classically as well since it is only encoded in the strongly amplified quadrature which, on its own, does not show any quantum signatures.

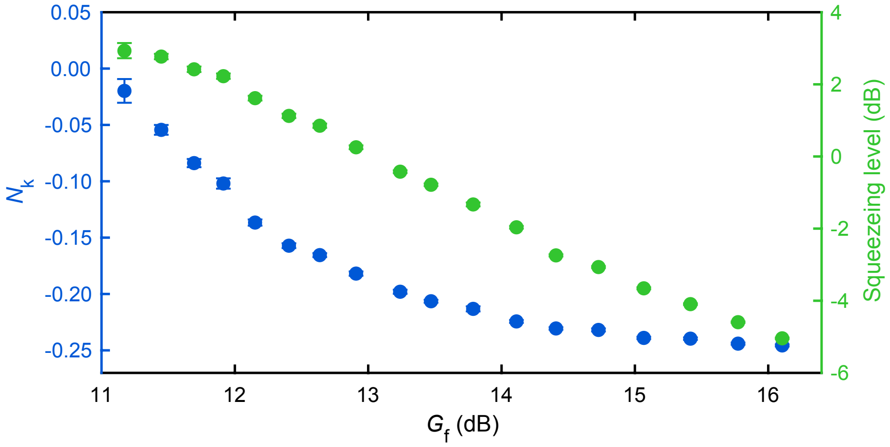

Supplementary Figure 5: Negativity kernel N_k (blue) and squeezing level (green) of the feedforward signal. The error bars are the standard error of the mean calculated from multiple repetitions of the protocol.

SUPPLEMENTARY NOTE 6: ENTROPY OF GAUSSIAN STATES

The von Neumann entropy $H(X) = -\operatorname{Tr}(\hat{\rho}_x \log \hat{\rho}_x)$ of a quantum state X with density matrix $\hat{\rho}_x$ is the quantum information analogue of the entropy used in thermodynamics (up to a factor of the Boltzmann constant k_B). For a Gaussian state, $H(X)$ can be calculated from its covariance matrix V. The von Neumann entropy of a single-mode Gaussian state is given by[14]

$$H(X) = f\left(\sqrt{\det V}\right) , \tag{18}$$

where $f(x) = \left(2x + \frac{1}{2}\right) \log\left(2x + \frac{1}{2}\right) - \left(2x - \frac{1}{2}\right) \log\left(2x - \frac{1}{2}\right)$.
For a two-mode Gaussian state AB, the covariance matrix can be expressed in the form

$$V = \begin{pmatrix} A & C \\ C^{\mathrm{T}} & B \end{pmatrix} , \tag{19}$$

where A, B and C are 2×2 matrices describing the local state A, local state B and cross-correlations between both parties, respectively. From V, one can calculate the two symplectic eigenvalues of the bipartite Gaussian state

$$\nu_\pm = \sqrt{\frac{\Delta \pm \sqrt{\Delta^2 - 4\det V}}{2}} , \tag{20}$$

where $\Delta = \det A + \det B + 2\det C$. The joint entropy of the whole bipartite state is given by

$$H(A, B) = f(\nu_+) + f(\nu_-) , \tag{21}$$

and the entropy of A conditioned on knowing B

$$H(A|B) = H(A, B) - H(B) \tag{22}$$

is called the conditional entropy. If both parties are correlated, knowledge about B will reveal information about A, and thus decrease its entropy, $H(A|B) < H(A)$.

SUPPLEMENTARY REFERENCES

[1] Yamamoto, T. *et al.* Flux-driven josephson parametric amplifier. *Appl. Phys. Lett.* **93**, 042510 (2008).
[2] Pogorzalek, S. *et al.* Hysteretic flux response and nondegenerate gain of flux-driven josephson parametric amplifiers. *Phys. Rev. Appl.* **8**, 024012 (2017).
[3] Tserkis, S. & Ralph, T. C. Quantifying entanglement in two-mode Gaussian states. *Phys. Rev. A* **96**, 062338 (2017).
[4] Fedorov, K. G. *et al.* Finite-time quantum entanglement in propagating squeezed microwaves. *Sci. Rep.* **8**, 6416 (2018).

[5] Menzel, E. P. *et al.* Dual-path state reconstruction scheme for propagating quantum microwaves and detector noise tomograph. *Phys. Rev. Lett.* **105**, 100401 (2010).
[6] Menzel, E. P. *et al.* Path entanglement of continuous-variable quantum microwaves. *Phys. Rev. Lett.* **109**, 250502 (2012).
[7] Eichler, C., Salathe, Y., Mlynek, J., Schmidt, S. & Wallraff, A. Quantum-limited amplification and entanglement in coupled nonlinear resonators. *Phys. Rev. Lett.* **113**, 110502 (2014).
[8] Xiang, S.-H., Wen, W., Zhao, Y.-J. & Song, K.-H. Evaluation of the non-Gaussianity of two-mode entangled states over a bosonic memory channel via cumulant theory and quadrature detection. *Phys. Rev. A* **97**, 042303 (2018).
[9] Scully, M. & Zubairy, M. *Quantum optics* (Cambridge University Press, Cambridge, 1997).
[10] Di Candia, R. *et al.* Quantum teleportation of propagating quantum microwaves. *EPJ Quan. Tech.* **2**, 25 (2015).
[11] Braunstein, S. L. & van Loock, P. Quantum information with continuous variables. *Rev. Mod. Phys.* **77**, 513 (2005).
[12] Paris, M. G. Displacement operator by beam splitter. *Phys. Lett. A* **217**, 78–80 (1996).
[13] Adesso, G. & Illuminati, F. Gaussian measures of entanglement versus negativities: Ordering of two-mode gaussian states. *Phys. Rev. A* **72**, 032334 (2005).
[14] Serafini, A., Illuminati, F. & Siena, S. D. Symplectic invariants, entropic measures and correlations of gaussian states. *J. Phys. B: At. Mol. Opt. Phys.* **37**, L21–L28 (2004).
